# Personalizing cognitive behavioral therapy for cancer-related fatigue using ecological momentary assessments followed by automated individual time series analyses: A case report series

**DOI:** 10.1016/j.invent.2021.100430

**Published:** 2021-07-14

**Authors:** Susan J. Harnas, Hans Knoop, Sanne H. Booij, Annemarie M.J. Braamse

**Affiliations:** a Amsterdam University Medical Centers, University of Amsterdam, Department of Medical Psychology, Cancer Center Amsterdam, Amsterdam Public Health Research Institute, Amsterdam, the Netherlands; b University of Groningen, Faculty of Behavioural and Social Sciences, Department of Developmental Psychology, Groningen, the Netherlands; c Center for Integrative Psychiatry, Lentis, Groningen, the Netherlands

**Keywords:** Individual time series analyses, Ecological momentary assessments, Personalization, Cognitive behavioral therapy, Cancer-related fatigue, Cancer survivors

## Abstract

**Introduction:**

A common approach to personalizing psychological interventions is the allocation of treatment modules to individual patients based on cut-off scores on questionnaires, which are mostly based on group studies. However, this way, intraindividual variation and temporal dynamics are not taken into account. Automated individual time series analyses are a possible solution, since these can identify the factors influencing the targeted symptom in a specific individual, and associated modules can be allocated accordingly. The aim of this study was to illustrate how automated individual time series analyses can be applied to personalize cognitive behavioral therapy for cancer-related fatigue in cancer survivors and how this procedure differs from allocating modules based on questionnaires.

**Methods:**

This study was a case report series (*n* = 3). Patients completed ecological momentary assessments at the start of therapy, and after three treatment modules (approximately 14 weeks). Assessments were analyzed with AutoVAR, an R package that automates the process of finding optimal vector autoregressive models. The results informed the treatment plan.

**Results:**

Three cases were described. From the ecological momentary assessments and automated time series analyses three individual treatment plans were constructed, in which the most important predictor for cancer-related fatigue was treated first. For two patients, this led to the treatment ending after the follow-up ecological momentary assessments. One patient continued treatment until six months, the standard treatment time in regular treatment. All three treatment plans differed from the treatment plans informed by questionnaire scores.

**Discussion:**

This study is one of the first to apply time series analyses in systematically personalizing psychological treatment. An important strength of this approach is that it can be used for every modular cognitive behavioral intervention where each treatment module addresses specific maintaining factors. Whether or not personalized CBT is more efficacious than standard, non-personalized CBT remains to be determined in controlled studies comparing it to usual care.

## Highlights

• Individual time series analyses can be used to systematically personalize CBT.
• Individual temporal dynamics between symptom and treatable factors can be studied.
• CBT based on individual time series analyses differs from usual modular approaches.
• Assessment during treatment enables the personalization of treatment duration.

## 1 Introduction

### 1.1 Background

There has been a repeated call for the personalization of evidence-based psychological interventions (U.S. Department of Health and Human Services NIoH, National Institute of Mental Health, 2008; U.S. Department of Health and Human Services NIoH, National Institute of Mental Health, 2015; U.S. Department of Health and Human Services NIoH, National Institute of Mental Health, 2020). Tailoring treatment to the characteristics and needs of the individual patient is assumed to improve treatment efficacy and patient adherence (U.S. Department of Health and Human Services NIoH, National Institute of Mental Health, 2008; U.S. Department of Health and Human Services NIoH, National Institute of Mental Health, 2015; U.S. Department of Health and Human Services NIoH, National Institute of Mental Health, 2020). One of the research directions so far has focused on personalizing psychological treatments by allocating treatment modules to individual patients (so called modular therapies) (Ng and Weisz, 2016). These modular therapies often consist of cognitive behavioral therapy (CBT), and are based on the theory that each module can function as a separate entity, targeting a cognitive or behavioral factor which is assumed to maintain or cause the targeted symptom. For example, a certain life-event may trigger a depression, but other factors (i.e. inactivity, dysfunctional cognitions) may maintain the depression. By measuring the presence of a factor thought to cause or maintain symptoms with a questionnaire, it can be determined which factors to target in treatment, and thereby, which modules to allocate to an individual patient. Thus, if a patient scores above a certain cut off point on a questionnaire at the start of treatment, the associated treatment module will be incorporated in the treatment plan (e.g. Abrahams et al. (2015); Gielissen et al. (2006); Prinsen et al. (2013); Poort et al. (2017)). As a result, the treatment plan of two patients can be very different.

Although personalized in some way, the norm scores used to determine if a treatment module should be allocated are based on cross-sectional nomothetic (i.e. group-based) research data. This way, interindividual differences are considered, but the intraindividual variation is not taken into account. It has been established previously that conclusions based on interindividual variation seldom can be generalized to intraindividual variation (Barlow and Nock, 2009; Molenaar and Campbell, 2009). The intraindividual variation in correlations between variables is often substantially larger than the between-persons variation (Fisher et al., 2018). Thus, when an association between two variables appears large based on between-persons variation, this association is often much weaker in an individual. For example, based on nomothetic research data there is an established association between fear and avoidance behavior. Avoidance behavior is mainly treated with exposure therapy (Fisher et al., 2018). However, the association between fear and avoidance behavior within an individual is more variable and often weaker. For some, the association between fear and avoidance will not even be present. Treating fear with exposure therapy in these individuals may not be successful. Furthermore, presence of a specific factor (e.g. avoidance behavior) thought to cause or maintain a symptom of interest on a group level does not automatically imply a causal relationship between the factor (e.g. avoidance behavior) and the symptom for a specific patient (e.g. fear). Thus, treating factors based on cut-off scores on questionnaires does not necessarily influence the symptom level in a specific patient, which often is the goal of treatment. Personalized treatment which targets only those factors that actually influence the symptom of interest in that specific individual seems to be more appropriate.

As opposed to nomothetic research, ideographic research methods focus on the individual patient level (Conner et al., 2009). A promising method for employing idiographic research is the use of ecological momentary assessments (EMA), also called experience sampling method (ESM) or diary methods (Conner et al., 2009; van der Krieke et al., 2015; Bolger et al., 2003). EMA is the repeated assessment of certain parameters (e.g. symptoms, experiences or activities), mostly multiple times a day, in order to collect real-time data in a natural setting (Conner et al., 2009; van der Krieke et al., 2015; Wenze and Miller, 2010). The main advantages of EMA are the reduction of memory bias, the ecological validity and the possibility to analyze the data on an individual level with time series analyses (Wenze and Miller, 2010). Individual time series analyses allow for elucidating individual symptom patterns over time and analyze temporal dynamics, providing an impression of putative causal associations (Wenze and Miller, 2010; Rosmalen et al., 2012). For example, Rosmalen et al. (Rosmalen et al., 2012) investigated temporal dynamics between physical activity and depression in patients who had experienced a myocardial infarction. Individual times series analyses showed different directions of causality. In one patient, increased activity levels predicted decreased depression scores. However, in two other patients increased depression scores predicted decreased activity scores. These differences could lead to different treatment advice, with a focus on either physical activation or the depressive symptoms. Thus, instead of using nomothetic-based cutoff scores on questionnaires to determine which treatment modules to assign, the allocation of treatment modules based on individual time series analyses might better reflect personalized psychological care.

A patient population which might benefit from more personalized care is the group of cancer survivors suffering from cancer-related fatigue. Cancer-related fatigue is one of the most common and debilitating symptoms among cancer survivors, affecting at least a quarter of survivors (Abrahams et al., 2016; Servaes et al., 2002). Cognitive behavioral therapy (CBT) for cancer-related fatigue has been proven effective in decreasing fatigue severity and improving patients’ functioning (Gielissen et al., 2006; Prinsen et al., 2015). The intervention was also effective in a blended format, in which internet-based treatment was combined with face-to-face and video sessions (Abrahams et al., 2017). In previous studies evaluating the efficacy of this intervention, the intervention had a modular approach, in which allocation of optional treatment modules occurred based on patients’ scores on questionnaires assessing maintaining factors (i.e. a cross-sectional nomothetic approach) (Abrahams et al., 2015; Gielissen et al., 2006; Prinsen et al., 2013; Poort et al., 2017). With individual time series analyses, temporal dynamics between cancer-related fatigue and its potential maintaining factors can be investigated, leading to a personalized treatment plan targeting only those maintaining factors actually influencing the targeted symptom.

An important challenge for time series analyses is its use in clinical practice. Analyzing time series data requires experience in this specific methodology and is time intensive, as analyses have to be conducted for each person separately (van der Krieke et al., 2015). A step forward in this area is the development of an application for performing automated time series analysis called AutoVAR (Emerencia et al., 2016). In short, AutoVAR is an R package with an easy-to use front-end web application that automates the process of finding optimal VAR models. By producing and comparing all possible valid models, it provides comprehensive and robust insights into the stability of results, while at the same time enabling rapid analysis and feedback on the EMA data. While this application is promising, it has yet to be determined how AutoVAR can be used to personalize psychological treatments in clinical practice (Emerencia et al., 2016). The aim of this case series report was to illustrate how automated individual time series analyses can be applied to personalize cognitive behavioral therapy (CBT) for cancer-related fatigue in cancer survivors and how this procedure differs from allocating modules based on questionnaires.

## 2 Methods

### 2.1 Study design

This study is designed as a case report series, in which the application of individual time series analyses with AutoVAR for personalizing CBT for cancer-related fatigue is illustrated. Personalization took place on the level of allocating treatment modules to individual patients.

The case report series consists of three case illustrations, in order to evaluate how the allocation of treatment modules can differ between patients based on automated individual time series. These three cases also illustrate how the allocation of treatment modules based on automated individual time series analyses differed from allocation based on questionnaires and cut-off scores. The three patients in this study were participants in a larger trial, the MATCH study. In the MATCH study, the use of EMA and time series analyses is part of a personalization ‘package’ which is evaluated and compared to care as usual (Harnas et al., under review), Dutch Trial Register (NTR): NL7481 (NTR7723)).

**Fig. 1:**
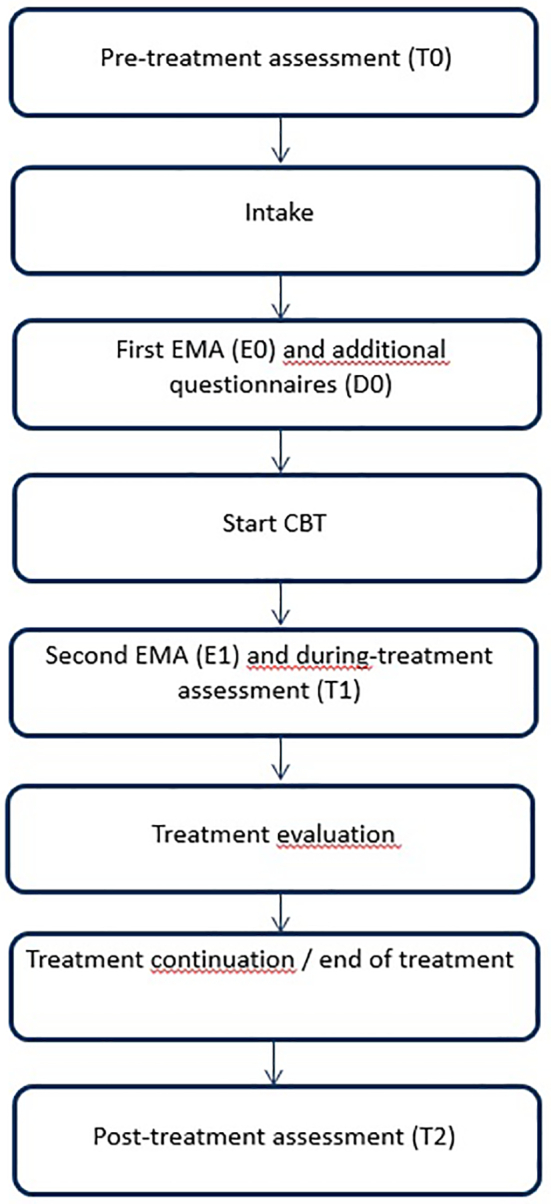
Study design.

In Fig. 1, an overview of the study design is provided. Patients completed assessments before treatment (T0), during treatment (T1) and after end of treatment (T2). These assessments consisted of the Checklist Individual Strength (CIS), subscale fatigue severity (Vercoulen et al., 1999; Vercoulen et al., 1994). After the first assessment (T0) and intake with the therapist, patients started with the first EMA during 14 consecutive days (E0). Patients also completed additional questionnaires measuring the presence of maintaining factors for cancer-related fatigue (D0). Automated individual time series analyses of the first EMA (E0) determined which optional treatment module to assign first, next to two mandatory modules. After completing these modules, patients started with the second EMA during 14 consecutive days (E1). If treatment was continued after the T1 assessment, the time series analyses of the second EMA (E1) determined which treatment modules to add or repeat.

### 2.2 Patients

We selected three cases for this study in August 2020. At that time, five patients 1) completed EMA at the start of treatment (E0) and after finalizing three treatment modules (E1); 2) completed CBT for cancer-related fatigue; and 3) completed fatigue severity assessments pre-treatment, during treatment and after end of treatment. Of these patients, we selected the first three cases that illustrated the most variation in how treatment plans can differ based on individual time series analyses with AutoVAR. As part of the inclusion criteria for the MATCH study, all three patients completed their primary, curative cancer treatment at least 6 months before referral, were ≥ 18 years old, were able to speak and read Dutch, had no disease activity at the time of inclusion in the study and were not currently receiving psychological or psychiatric treatment. Also, all patients filled out screening questionnaires before participation in the MATCH-study, in which they reported clinically relevant levels of fatigue (Checklist Individual Strength (CIS), cutoff ≥35 on the fatigue severity subscale) (Vercoulen et al., 1999; Vercoulen et al., 1994), and experienced functional impairments (Work and Social Adjustment Scale (W&SAS), cutoff ≥10 (Mundt et al., 2002; Mataix-Cols et al., 2005).

### 2.3 Evidence-based CBT for cancer-related fatigue

According to the blended, evidence-based protocol for cancer-related fatigue, patients started treatment with setting their personal treatment goals, and ends treatment with evaluating and realizing these goals (Abrahams et al., 2015). The treatment further includes the following six treatment modules: 1) Sleep-wake rhythm, 2) Activity pattern, 3) Helpful thinking, 4) Coping with cancer and cancer treatment, 5) Fear of cancer recurrence and 6) Social support. For a description of the content of each of the treatment modules, we refer to the paper of Abrahams et al. (2015). In the evidence-based protocol, modules 4-6 are optional. At baseline it was decided which optional modules were relevant for each patient, based on questionnaires scores.

For this study, we divided the six treatment modules into two mandatory modules and four optional modules. The two mandatory modules were: 1) sleep-wake rhythm and 2) activity regulation. The four optional modules were: 1) coping with cancer and cancer treatment, 2) fear of cancer recurrence, 3) helpful thinking, and 4) social support. This way, every patient was exposed to at least two modules in which factors are targeted that in previous research appeared to mediate the effect of CBT on fatigue, but also enough room was left for personalization (van den Akker et al., 2018). According to this approach, patients in this study started with sleep-wake rhythm, activity pattern and one optional module. Two patients received CBT in a blended format, which means a combination of face-to-face sessions and web-based treatment. One patient received CBT in an online format, which means only the web-based treatment. For the web-based treatment, we used a secure web-based environment (Minddistrict; www.minddistrict.com), in which patients work through the different treatment modules. All treatment modules consisted of three parts: psycho-education, assignments and an evaluation (Abrahams et al., 2015). As every treatment module consisted of multiple assignments, a patient needed several therapy sessions to complete a treatment module. The therapy took 21-29 weeks to complete and consisted of 12 to 16 sessions with their therapist, depending on the treatment plan of the individual patient. All patients started treatment with 2 face-to-face sessions and ended treatment with a face-to-face session. The blended treatment consisted of 4 extra face-to-face sessions and 5-9 online sessions. The online treatment, consisted of one telephone session and 8-12 online sessions, besides the 3 face-to-face sessions at the start and end of treatment. In a face-to-face session, assignments from the different treatment modules were discussed and/or a new treatment module was introduced. The online sessions were structured in the same manner, but the sessions were asynchronous, took place via secured e-mail through the Minddistrict platform and consisted of written feedback from the therapist. Providing online feedback took therapists approximately 20-30 min per patient per session and a face-to-face sessions approximately 45 min. During treatment, the therapist had the option to add one or two video sessions, if deemed necessary. A video session was structured in the same way as the face-to-face session, but the session took place through a video call in our secured web-based environment (Minddistrict; www.minddistrict.com).

### 2.4 Personalized CBT for cancer related fatigue

For personalizing CBT for cancer-related fatigue, we used automated individual time series analyses to determine which optional treatment module to assign first. We used the first ecological momentary assessment period for these analyses (E0). The strongest predictor for fatigue determined which optional module to add to the treatment plan, as every predictor was associated with a specific treatment module. If individual time series analyses showed no predictors, the questionnaires measuring the maintaining factors (see Table 1 and paragraph ‘2.5. Measurements’) were used to determine which optional module to assign first. This way, every patient was exposed to the same number of treatment modules before the second EMA (E1), i.e. the two mandatory modules and one optional module. After finalizing the three treatment modules, patients completed the second EMA (E1) and the T1 assessment (Fig. 1). At the first follow-up assessment (T1), fatigue severity was assessed again. If the score on the subscale fatigue severity was <35, the patients was advised to stop treatment. If the score on the subscale fatigue severity of the Checklist Individual Strength was ≥35 (Vercoulen et al., 1999; Vercoulen et al., 1994), the advice was given to continue treatment. If the patient agreed to continue treatment, automated individual time series analyses of the second EMA (E1) determined which treatment modules to add or repeat. If it was advised to end treatment, but the patient expressed a preference for continuing treatment, automated individual time series analyses of the second EMA (E1) was used to determine which treatment modules to add or repeat.

**Table 1:** Overview of the maintaining factors targeted in the optional treatment modules in personalized CBT. A score in bold indicates that the score is above the cut-off score.

Treatment module	Targeted maintaining factor	Measured with the following questionnaire(s)	Subscale(s)	Cut-off score	Patient A	Patient B	Patient C
Coping with cancer and cancer treatment	Poor coping with cancer and cancer treatment	Impact of Event Scale (IES) (Brom and Kleber, 1985; van der Ploeg et al., 2004)	Avoidance	≥ 10	**24**	0	**28**
			Intrusion	≥ 10	**19**	0	**23**
Fear of cancer recurrence	Fear of cancer recurrence	Cancer Worry Scale (CWS) (Custers et al., 2018)	–	≥ 10	**16**	8	**17**
Social support	Low social support	van Sonderen Social Support List (SSL) (shortened version) (Poort et al., 2017; van Sonderen, 1993; van Sonderen and Ormel, 1997)	Negative Interactions (SSL-N)	≥ 10	9	7	**10**
			Discrepancies (SSL-D)	≥ 14	12	10	10
Helpful thinking	Dysfunctional cognitions regarding fatigue	Illness Management Questionnaire (IMQ) (Ray et al., 1993; Andrykowski et al., 2005)	Focusing on symptoms (IMQ-FS)	≥ 30	**43**	**30**	**42**
		Fatigue Catastrophizing Scale (FCS) (Jacobsen et al., 2004)	–	≥ 16	**41**	**16**	**27**
		Self-efficacy Scale (SES) (Gielissen et al., 2007)	–	≤ 19	**17**	22	21
Number of modules indicated according to questionnaires:					3	1	4

### 2.5 Measurements

#### 2.5.1 Fatigue severity

The primary outcome in this case report series was fatigue severity, measured with the subscale fatigue severity of the Checklist Individual Strength (CIS) (Vercoulen et al., 1999; Vercoulen et al., 1994). The subscale consists of 8 items, which can be rated on a 7-point Likert scale. Total scores on the subscale fatigue severity range from 8 to 56, with a score of 35 points or higher indicating severe fatigue. The CIS-fatigue has previously been used in intervention studies, has been proven reliable and sensitive to change, and has good discriminative validity (Gielissen et al., 2006; Goedendorp et al., 2010; Beurskens et al., 2000). The primary outcome was assessed pre-treatment (T0), during treatment (T1) and after end of treatment (T2).

#### 2.5.2 EMA-survey

The EMA-survey consisted of 13 questions in which the following dimensions were assessed: fatigue, depression, fear of cancer recurrence, physical activity, mental activity, social activity, focus on fatigue, catastrophizing, powerlessness, self-efficacy, intrusion, avoidance and lack of understanding. The EMA survey contained items of the questionnaires mentioned in Table 1. The wording of the selected items was adjusted to reflect the nature of EMA. If possible, we selected the questionnaire items with high factor loadings. If this was not possible, we discussed the options with a team of experienced researchers and clinical psychologists in order to decide which item (and which wording) best reflected the dimension until consensus was reached. During the first and last EMA of the day we asked 4 additional questions regarding sleep during the day (at the end of the day) and sleep during the night (first EMA of the day). As the sleep items were assessed less frequently than the other items, the sleep items were not included in the individual time series analyses. Also, the depression item was not included in the individual time series analyses as this is not regarded a potential maintaining factor for fatigue in the treatment protocol. The EMA survey is added as supplementary material (Supplement A).

#### 2.5.3 Additional questionnaires

All patients completed additional questionnaires after the intake with the therapist (see ‘D0’ in Fig. 1). With these questionnaires it was evaluated which maintaining factors were present in an individual patient by comparing the score on the questionnaire to a cut-off score (see Table 1). According to the evidence-based protocol for cancer-related fatigue, a treatment module should be offered to a patient if the score on the accompanying questionnaire is above the cut-off (Abrahams et al., 2015; Gielissen et al., 2006; Prinsen et al., 2013; Poort et al., 2017).

### 2.6 Ecological momentary assessments (EMA)

#### 2.6.1 EMA protocol

EMA were offered 5 times a day during 14 consecutive days. Intervals between the assessments were fixed, which meant that every three hours patients needed to complete an assessment. Although a fixed time schedule may negatively impact ecological validity, it was adopted to accommodate the application of vector autoregressive modelling, which requires (about) equally spaced intervals (see 2.6.2.2 for more information). The exact time points were adapted to a patients’ sleep-wake schedule. Patients received a text message on their mobile phone, in which a link to a questionnaire was offered. Patients needed to click on the link to open the questionnaire in the web browser. The link to the questionnaire was valid for one hour. If patients did not complete the assessment within 30 min, a reminder text message was sent. If the patients were not able to complete the questionnaire within one hour, the assessment was identified as a missing measurement.

#### 2.6.2 Data analyses

The time series of each patient was analyzed by the primary researcher (SH) directly after obtaining the complete measurements, at the start of treatment (E0) and during treatment (E1).

##### 2.6.2.1 Data preparation

Before conducting the analyses, the mean square successive difference (MSSD) of the variables was checked. To ensure sufficient variability within each variable and, as such, increase the probability of finding a valid VAR model, variables with an MSSD of 50 or less were not included in the AutoVAR analyses (Krieke et al., 2016). Although the primary reason for excluding these variables was statistical, variables with low variability are also theoretically less interesting, as within the VAR framework short-term dynamics around an equilibrium are assessed. From that perspective, a non-fluctuating variable is unlikely to be an important predictor of fluctuations in another variable, and vice versa. If all variables showed sufficient variability, a maximum of 12 variables could be included in the analyses. Next, the data was checked for missing measurements. The missing measurements were imputed using the Amelia II package in R (Honaker et al., 2011). Amelia II is a multiple imputation method suitable for time-series data, that uses expectation-maximization with bootstrapping. Each participant's data was imputed separately, providing 5 imputed datasets per participant. To perform analyses, these datasets were combined by averaging. Predictor variables were lags of all the variables under study as well as a second-order polynomial of time. To meet its assumption of normally distributed variables, the variables were inspected prior to imputation and log or power transformed in case of right- or left-skewed variables, respectively.

##### 2.6.2.2 Vector autoregressive modelling

With vector autoregressive modelling, temporal dynamics between two or more time series can be investigated. In addition, by separating the dynamic part of the model (i.e. the relationships between the time-lagged values of the variables) from the simultaneous part (i.e. the relationships between the contemporaneous variables), the model enables inferences about the temporal order of the effects, also known as Granger causality (Rosmalen et al., 2012; Brandt and Williams, 2006). Specifically, a variable Y is said to “Granger cause” Z if past values of Y improve the prediction of Z, and more so than past values of Z alone can do (Rosmalen et al., 2012; Lütkepohl, 2006). Hence, temporal associations between (maintaining) factors and a symptom of interest (in this case, cancer-related fatigue) can be established. Individual time series were analyzed with “AutoVAR”, an application in which time series can be analyzed automatically with vector autoregressive modelling (van der Krieke et al., 2015; Emerencia et al., 2016; Brandt and Williams, 2006). We used the package AutoVAR in R, version 1.2.1335 (Emerencia et al., 2016). For those unfamiliar with R, a front end website is also available (www.autovar.nl). Its main functionality is producing a list of VAR models that do not invalidate the model assumptions of VAR (i.e. white noise assumption, stationarity, homoscedasticity and normality of the residuals). AutoVAR can summarize over the models to provide insight into, e.g., significant Granger causalities present in the data set. For more details about the AutoVAR procedure, please see Emerencia et al. (2016). In this study, a maximum lag length of 2 (e.g. a change in one variable leads to a change in another variable approximately 6 h later) was applied and initially only associations were analyzed between the cancer-related fatigue and each different maintaining symptom separately.

##### 2.6.2.3 Personalizing the treatment plan based on EMA and time series analyses

After the first ecological momentary assessment period we determined which optional treatment module to assign to the patient, besides the first two mandatory treatment modules. For this, we identified the most relevant maintaining factor of cancer-related fatigue in the individual patient, associated with one of the optional treatment modules. The most relevant maintaining factor was identified as the factor that Granger caused cancer-related fatigue in most of the valid VAR models (highest percentage) that were produced by AutoVAR. Accordingly, the associated optional treatment module was added to the treatment plan. If two or more maintaining factors, associated with the optional modules, showed an equal percentage, the factors were simultaneously analyzed in one model to determine the strongest maintaining factor. If treatment continued after the first follow-up assessment (T1), the second ecological momentary assessment determined which treatment module to repeat or add to the treatment plan. For this, we identified which maintaining factors (still) Granger caused cancer-related fatigue. Associated treatment modules were added to the treatment plan or repeated.

## 3 Results: case illustrations

### 3.1 Patient A

Patient A was a 60-year old woman, 18 months after end of treatment for breast cancer, referred for psychological treatment because of cancer-related fatigue. She underwent treatment with curative intent consisting of neoadjuvant chemotherapy, a mastectomy, adjuvant radiotherapy, and adjuvant hormonal therapy. At the time of referral she still received hormonal therapy. At baseline (T0), before intake and start of treatment, patient A had a score of 47 on the subscale fatigue severity of the Checklist Individual Strength (CIS), indicating presence of severe fatigue (see Table 2).

**Table 2:** Scores of patient A, B and C pre-treatment (T0), during treatment (T1) and after end of treatment (T2).

Score on the subscale fatigue of the Checklist Individual Strength (CIS) Cut-off value ≥ 35			
Measurement	Patient A	Patient B	Patient C
T0	47	44	41
T1	16	21	21
T2	16	16	37

#### 3.1.1 First EMA (E0)

**Fig. 2:**
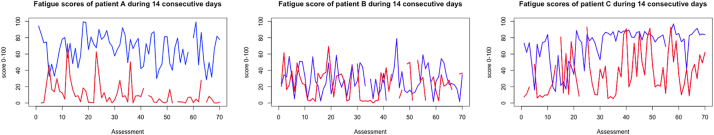
Fatigue scores of patient A, B and C during 14 consecutive days in the first and second ecological momentary assessments (EMA). Blue line = first EMA (E0), Red line = second EMA (E1).

**Fig. 3:**
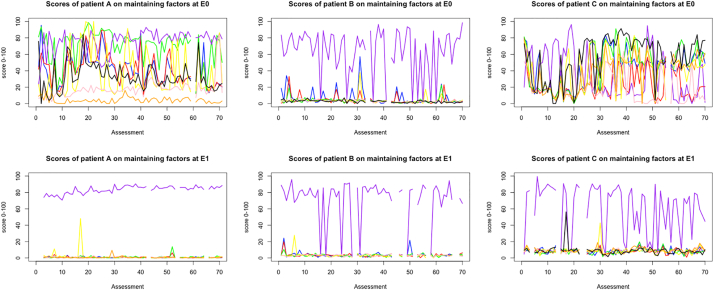
Scores on maintaining factors of patient A, B and C during 14 consecutive days in the first and second ecological momentary assessments (EMA). Blue line = focus on fatigue, red line = catastrophizing, green = powerlessness, purple = self-efficacy, intrusion = yellow, avoidance = pink and lack of social understanding = black. Note: for all maintaining factors except self-efficacy, a low score means less burden.

**Fig. 4:**
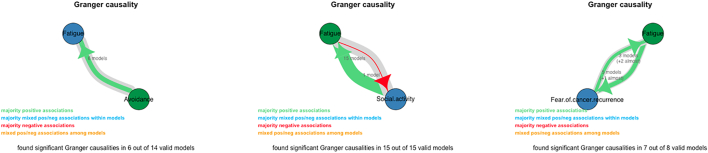
Part of the output shown by the application AutoVAR, illustrating the Granger causality summary graph of the strongest predictor of patient A, B and C at E0, respectively.

After the intake, Patient A completed the first EMA. She completed 69 of the total of 70 assessments. In Fig. 2 the fatigue scores of patient A during the first EMA is plotted (E0, blue line). In Fig. 3 the scores on the maintaining factors of patient A at E0 are shown. One variable had a mean successive difference (MSSD) of less than 50. Based on the analyses, four factors Granger caused fatigue. These four factors were associated with two optional treatment modules. The factor that predicted fatigue in the most valid VAR models was avoidance of things or situations that reminded her of cancer and cancer treatment. Higher scores on the avoidance item predicted higher scores on the fatigue item (Fig. 4).

#### 3.1.2 Treatment plan after E0

**Fig. 5:**
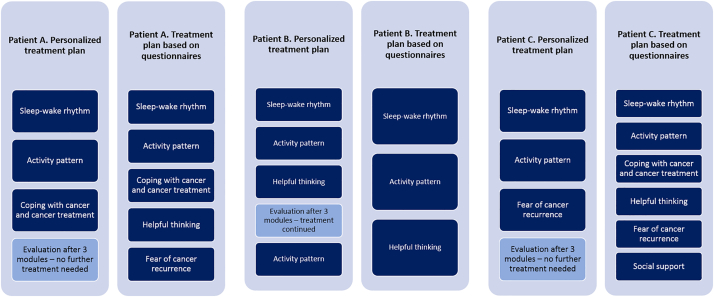
Personalized treatment plan versus treatment plan based on questionnaires for patient A, B and C.

Based on the first EMA, we concluded that avoidance of situations or things that made her think about cancer was the most relevant maintaining factor of fatigue in this patient. The associated treatment module (‘Coping with cancer and cancer treatment’) was added to the treatment plan (Fig. 5). A comparison with the results from the additional questionnaires (D0 in Fig. 1) (see Table 1) reveals that, based on these scores, three optional modules were indicated: (1) Coping with cancer and cancer treatment, (2) Fear of cancer recurrence and (3) Helpful thinking (Fig. 5).

#### 3.1.3 Follow-up assessment

After finalizing the three treatment modules according to the personalized treatment plan, patient A completed the Checklist Individual Strength (CIS) again. She showed a score of 16 on the subscale fatigue severity of the Checklist Individual Strength (CIS) (see Table 1), indicating absence of severe fatigue. Patient A also completed the second EMA. She completed 66 of the total of 70 assessments. In Fig. 2 the fatigue scores of patient A during the second EMA is plotted (E1, red line). In Fig. 3 the scores on the maintaining factors of patient A at E1 are shown. Seven variables had a mean successive difference (MSSD) of less than 50. Bases on the analyses, one factor Granger caused fatigue. This factor was associated with the mandatory treatment module ‘activity pattern’ (see Fig. 5). Higher scores on the physical activity item predicted higher scores on the fatigue item.

#### 3.1.4 Treatment plan after E1

At T1, patient showed a score of 16 on the subscale fatigue severity of the Checklist Individual Strength (CIS) (see Table 2), indicating absence of severe fatigue. Therefore, it was advised that CBT could be ended. Patient A agreed with this advice. Therefore, patient A finished treatment by evaluating the realization of her goals (Fig. 5). She had realized all of her goals except for increasing her physical activity by gardening – which she was still working on. So she recognized the existing association between physical activity and fatigue. However, she felt she was able to manage this by herself, using the tools for gradually increasing activity levels she had received during treatment. In agreement with the patient, treatment was ended. In total, treatment consisted of 12 sessions (face-to-face or online, no use of additional video sessions) spread over 21 weeks. After completing treatment, patient A completed the Checklist Individual Strength (CIS) once again. She still had a score of 16 on the subscale fatigue severity of the Checklist Individual Strength (CIS) (see Table 1), indicating absence of severe fatigue.

### 3.2 Patient B

Patient B was a 56-year old woman, 18 months after end of treatment for breast cancer, referred for psychological treatment because of cancer-related fatigue. She underwent treatment with curative intent consisting of a partial mastectomy, adjuvant chemotherapy, adjuvant radiotherapy and adjuvant hormonal therapy. At the time of referral patient still received hormonal therapy. At baseline (T0), before intake and start of treatment, patient B had a score of 44 on the subscale fatigue severity of the Checklist Individual Strength (CIS), indicating presence of severe fatigue (see Table 2).

#### 3.2.1 First EMA (E0)

Patient B completed the first EMA after intake. Patient B completed 68 of the 70 offered assessments. In Fig. 2 the fatigue scores of patient B during the first EMA is plotted (E0, blue line). In Fig. 3 the scores on the maintaining factors of patient B at E0 are shown. Six variables had a mean successive difference (MSSD) of less than 50. Based on the analyses, three variables Granger caused fatigue. These variables were: social activity, mental activity and physical activity. The three factors were not associated with one of the four optional treatment modules. The factor that predicted fatigue in the most valid VAR models (highest percentage) was social activity (see association in Fig. 4). Higher scores on the social activity item predicted higher scores on the fatigue item (Fig. 4).

#### 3.2.2 Treatment plan after E0

As the individual time series analyses showed that no factors associated with one of the optional treatment modules Granger caused fatigue, we used the questionnaires to determine which optional module to assign first. Based on the questionnaires, one optional module was indicated for patient B (see Table 1). This treatment module was helpful thinking. Therefore, this optional treatment module was assigned to patient B (Fig. 5).

#### 3.2.3 Follow-up assessment

After finalizing the three treatment modules, patient B completed the Checklist Individual Strength (CIS) again. Patient scored 21 on the subscale fatigue severity of the Checklist Individual Strength (CIS) (see Table 1), indicating absence of severe fatigue. Patient B also completed the second EMA (62 of the 70 assessments). In Fig. 2 the fatigue scores of patient B during the second EMA are plotted (E1, red line). In Fig. 3 the scores on the maintaining factors of patient B at E1 are shown. Eight variables showed a mean successive difference (MSSD) of less than 50. Based on the analyses, three factors Granger caused fatigue. These factors were all associated with the mandatory treatment module ‘activity pattern’ (see Fig. 5). Higher scores on the activity items predicted higher scores on the fatigue item.

#### 3.2.4 Treatment plan after E1

At T1, patient B showed a score of 21 on the subscale fatigue severity of the Checklist Individual Strength (CIS) (see Table 2), indicating absence of severe fatigue. Therefore, it was advised that CBT could be ended. However, patient B preferred to continue with treatment. Therefore, treatment was continued by repeating the ‘activity pattern’ module (Fig. 5). After repeating this module, treatment was ended. In total, treatment consisted of 15 sessions (face-to-face or online, 1 video session) spread over 25 weeks. After completing treatment, patient B completed the Checklist Individual Strength (CIS) once again. She had a score of 16 on the subscale fatigue severity of the Checklist Individual Strength (CIS) (see Table 1), indicating absence of severe fatigue.

### 3.3 Patient C

Patient C was a 50-year old woman, 34 months after end of treatment for breast cancer, who applied for participation in the MATCH study because of cancer-related fatigue. She underwent treatment with curative intent consisting of a full mastectomy and adjuvant hormonal therapy. At the time of referral patient C still received hormonal therapy. At baseline (T0), before intake and start of treatment, patient C had a score of 41 on the subscale fatigue severity of the Checklist Individual Strength (CIS), indicating presence of severe fatigue (see Table 2).

#### 3.3.1 First EMA (E0)

After intake, Patient C completed the first EMA. She completed 69 of the total of 70 assessments. In Fig. 2 the fatigue scores of patient C during the first EMA are plotted (E0, blue line). In Fig. 3 the scores on the maintaining factors of patient C at E0 are shown. All variables had a mean successive difference (MSSD) of more than 50. Based on the analyses, one factor Granger caused fatigue. This factor was associated with one of the optional treatment modules (see Fig. 5). Higher scores on the fear of cancer recurrence item predicted higher fatigue scores (Fig. 4).

#### 3.3.2 Treatment plan after E0

Based on the first EMA, we added the optional treatment module ‘Fear of cancer recurrence’ to the treatment plan (Fig. 5). A comparison with the results from the additional questionnaires (D0 in Fig. 1) (see Table 1) reveals that, based on these scores, all four optional modules were indicated: (1) Coping with cancer and cancer treatment, (2) Fear of cancer recurrence, (3) Helpful thinking and (4) Social support (Fig. 5).

#### 3.3.3 Follow-up assessment

After finalizing the three treatment modules, patient C completed the Checklist Individual Strength (CIS) again. She scored 21 on the subscale fatigue severity of the Checklist Individual Strength (CIS) (see Table 1), indicating absence of severe fatigue. Patient C also completed the second EMA. She completed 66 out of the total of 70 assessments. Fig. 2 shows the fatigue scores of patient C during the second EMA (E1, red line). In Fig. 3 the scores on the maintaining factors of patient C at E1 are shown. Six variables had a mean successive difference (MSSD) of less than 50. Based on the analyses, three factors Granger caused fatigue. These factors were associated with the mandatory treatment module ‘activity pattern’ and the two optional treatment modules ‘fear of cancer recurrence’ and ‘helpful thinking’ (see Fig. 5). The association from fear of cancer recurrence to fatigue was still positive, indicating that higher scores on the fear of cancer recurrence item predicted higher fatigue scores.

#### 3.3.4 Treatment plan after E1

At T1, patient showed a score of 21 on the subscale fatigue severity of the Checklist Individual Strength (CIS) (see Table 2), indicating absence of severe fatigue. Therefore, it was advised that CBT could be ended. Patient C agreed with this advice. Therefore, patient C finished treatment by evaluating the realization of her goals (Fig. 5). In total, treatment consisted of 12 sessions (face-to-face or online, no use of additional video sessions) spread over 21 weeks. After end of treatment, patient completed the Checklist Individual Strength (CIS) once again. She had a score of 37 on the subscale fatigue severity of the Checklist Individual Strength (CIS) (see Table 1), indicating recurrence of severe fatigue. Patient C provided us with additional information that certain life-events had led to an increase in her fatigue score; she did not experience this as a relapse of the cancer-related fatigue.

## 4 Discussion

With this case series report we illustrated how individual time series analyses with AutoVAR can be used to personalize CBT for cancer-related fatigue. As this is one of the first attempts to apply this method for personalizing treatment plans in clinical practice, we expect these results to be informative for clinicians as well as researchers in the field. Based on the three cases in this study, application of this method seems feasible in clinical practice as patients were willing and able to complete the EMA, and individual time series analyses with AutoVAR resulted in relevant associations between treatable maintaining factors and cancer-related fatigue. Altogether, this study has several important findings for the personalization of psychological treatment.

The results from this study confirm that presence of maintaining factors as measured with questionnaires, not necessarily means that these factors predict the targeted symptom (in this case, cancer-related fatigue). In patient C all maintaining factors associated with the optional treatment modules were present according to the questionnaire scores. However, individual time series analyses with AutoVAR showed that only one of these factors was actually predicting a change in the fatigue of this patient in daily life. In patient B, one optional treatment module was indicated based on the scores on the questionnaires. Based on individual time series analyses with AutoVAR however, no optional treatment modules were indicated as no predictors associated with these modules were identified. In patient A, three optional modules were indicated based on the questionnaires. However, individual time series analyses with AutoVAR showed that two of the three optional modules were indicated as the associated maintaining factors predicted changes in the fatigue. The differences between (mean-level) presence of supposed maintaining factors and the presence of factors predicting changes in the symptom over time suggest the merit of an individualized (as opposed to a group-based) treatment approach. Identifying those factors influencing individual patients’ symptoms seems a premise for truly personalizing CBT. With personalizing CBT, the ultimate goal is to design an individualized treatment plan which is most effective and efficient for that specific patient. Identifying the most relevant maintaining factors at the start of treatment and adding associated treatment modules to the treatment plan could be a promising method for this. The assumption is that, this way, per patient the most important factor influencing the cancer-related fatigue is targeted first. As illustrated in Fig. 3, this approach led to the improvement in the other maintaining factors that were present, while these not being directly targeted during treatment. At a fundamental level, this approach differs from the nomothetic-based approach in the position of the underlying theoretical model. In a nomothetic-based approach, the theoretical model is the main guidance in making treatment decisions, i.e. the patient is fit into the model. The idiographic-based approach as applied in this study has the theoretical model as starting point, and adds the person-specific associations and dynamics resulting from the ecological momentary assessments, so that case conceptualization is truly more individualized (Fisher and Boswell, 2016).

Adding an ‘in-between’ assessment after a couple of modules instead of only evaluating the treatment effect at the end of treatment enables the personalization of treatment duration. In this study, we used symptom level combined with automated individual time series analyses and patient preferences to make this decision. As we had no in-between assessment of maintaining factors with questionnaires, it is not possible to conclude whether the use of automated individual time series for selecting appropriate treatment modules leads to a more efficient (i.e. shorter) treatment plan compared with a treatment plan based on questionnaire scores. We observed, however, that the in-between assessment led to a shorter treatment duration when compared to the original treatment protocol in two of our three cases. As criteria for (dis)continuing treatment, we used the cutoff score for severe fatigue on the CIS (Vercoulen et al., 1999; Vercoulen et al., 1994) and patients’ needs. The fatigue severity score of patient A and C had declined to a non-clinical level at the T1 assessment. Therefore, we advised to end treatment, and the two patients agreed with this advice. Although the fatigue severity score of the third patient (patient B) also declined to a non-clinical level at the T1 assessment, she expressed a wish for treatment continuation as she had not completed her treatment goals yet. In our view, this observation stresses the importance of not only taking cut-off scores and presence of relevant associations with maintaining cognitive behavioral factors into account, but also considering individual preferences and a need for care. In the case of patient B, taking her preference for treatment continuation seriously and continuing treatment according to results of the individual time series analyses of the follow-up EMA, she showed a further decrease of fatigue severity. For patient C one could wonder whether discontinuation of treatment happened too soon, as her fatigue score at the T2 assessment returned to a clinical level. Further treatment could have focused on the relevant associations that resulted from her second time series analyses. However, it is to be expected that people with normal fatigue also show some associations of certain cognitive behavioral factors (e.g. increased activity, or less sleep) with fatigue, without needing treatment as long as the fatigue level is in the normal range. The aforementioned shows that the decision to stop treatment is a complex one, based on multiple factors, among which are symptom level and presence of treatable cognitive behavioral factors, and also individual preferences, individual circumstances and findings with respect to the course of fatigue following treatment. Ultimately, our experiences and the future findings of the RCT provide valuable input for the development of more strict criteria for (dis)continuing treatment.

There are some challenges with regard to using EMA and time series analyses in clinical practice. The analyses, even with AutoVAR, require sufficient knowledge to understand the output, and basic knowledge about the underlying techniques and assumptions, especially in case of errors. Probably the application of these techniques is not suitable for every therapist. Further, it is a time intensive procedure for patients. In case of too many missing assessments, time series analyses are not possible, and as such, creating a treatment plan based on these analyses fails. This requires motivated patients, well-working equipment (e.g. smartphones, Wi-Fi connection), and strict monitoring by, for example, a psychological assistant. Also personal sensing, by means of smartphones or wearables, holds great promise as a method for assessing activities, sleep, and emotions or mood, with minimal effort from the user. Matching behavioral interventions to these assessments could be a valuable next step in personalized treatment (Mohr et al., 2017).

The EMA survey in this specific study was constructed with items from validated questionnaires, if possible with high factor loadings. The validity of this specific survey has not been formally evaluated (Degroote et al., 2020). It would be interesting to evaluate the correlations between the items of the EMA survey and the actual whole scale of the validated questionnaires to further investigate the validity of the EMA-survey. Also, recently a guideline was developed with a new approach to determine content validity of patient outcome measures (Terwee et al., 2018). Future studies could follow this guideline.

There are also some challenges to the modelling of the data at hand. To determine which factor is the strongest predictor for cancer-related fatigue in a specific individual, we looked within the valid models which variable was most often a Granger causal factor for fatigue. However, sometimes a limited number of valid models appeared. This could suggest that the data at hand is better suitable for other types of (VAR) analyses. A more advanced technique, such as time-varying VAR (Haslbeck et al., 2020), for example, might be indicated, which is able to handle non-stationary data. In addition, following the assumptions of VAR models, we adopted a fixed sampling scheme with equidistant intervals, whereas as a (semi)-random scheme might have increased ecological validity (Verhagen et al., 2016). Application of continuous-time models would allow for unequally spaced sampling schemes (Ryan et al., 2018; Oravecz et al., 2016). Ideally, these different techniques would be incorporated in one package that automates the process of choosing the best statistical technique for the data at hand.

In closing, this innovative study is one of the first to apply EMA and individual time series analyses in systematically personalizing psychological treatment plans on the level of the individual. This approach focuses on individual models and enables the personalization of psychological treatment plans in a systematic way. For reasons of clarity, the method is illustrated with three cases of cancer survivors suffering from cancer-related fatigue. An important strength of this approach is that it can be used for every modular CBT where maintaining or causal cognitive behavioral factors are targeted in order to decrease psychological symptoms. Whether or not personalized CBT is more efficacious than standard, non-personalized CBT remains to be determined in controlled studies comparing it to usual care.

The following are the supplementary data related to this article.Supplementary A Supplement A - EMA survey Supplementary A

## Declaration of competing interest

The authors declare that they have no known competing financial interests or personal relationships that could have appeared to influence the work reported in this paper.

## References

[bb0005] Abrahams H.J., Gielissen M.F., Goedendorp M.M., Berends T., Peters M.E., Poort H. (2015). A randomized controlled trial of web-based cognitive behavioral therapy for severely fatigued breast cancer survivors (CHANGE-study): study protocol. BMC Cancer.

[bb0010] Abrahams H.J., Gielissen M.F., Schmits I.C., Verhagen C.A., Rovers M.M., Knoop H. (2016). Risk factors, prevalence, and course of severe fatigue after breast cancer treatment: a meta-analysis involving 12 327 breast cancer survivors. Ann. Oncol..

[bb0015] Abrahams H.J.G., Gielissen M.F.M., Donders R.R.T., Goedendorp M.M., van der Wouw A.J., Verhagen C. (2017). The efficacy of internet-based cognitive behavioral therapy for severely fatigued survivors of breast cancer compared with care as usual: a randomized controlled trial. Cancer.

[bb0020] Andrykowski M.A., Schmidt J.E., Salsman J.M., Beacham A.O., Jacobsen P.B. (2005). Use of a case definition approach to identify cancer-related fatigue in women undergoing adjuvant therapy for breast cancer. J. Clin. Oncol..

[bb0025] Barlow D.H., Nock M.K. (2009). Why can’t we be more idiographic in our research?. Perspect. Psychol. Sci..

[bb0030] Beurskens A.J.H.M., Bultmann U., Kant I., Vercoulen J.H.M.M., Bleijenberg G., Swaen G.M.H. (2000). Fatigue among working people: validity of a questionnaire measure. Occup. Environ. Med..

[bb0035] Bolger N., Davis A., Rafaeli E. (2003). Diary methods: capturing life as it is lived. Annu. Rev. Psychol..

[bb0040] Brandt P.T., Williams J.T. (2006). Multiple Time Series Models.

[bb0045] Brom D., Kleber R.J. (1985). De schok verwerkings lijst. Ned. Tijdschr. Psychol..

[bb0050] Conner T.S., Tennen H., Fleeson W., Barrett L.F. (2009). Experience sampling methods: a modern idiographic approach to personality research. Soc. Personal. Psychol. Compass.

[bb0055] Custers J.A.E., Kwakkenbos L., van de Wal M., Prins J.B., Thewes B. (2018). Re-validation and screening capacity of the 6-item version of the cancer worry scale. Psychooncology.

[bb0060] Degroote L., DeSmet A., De Bourdeaudhuij I., Van Dyck D., Crombez G. (2020). Content validity and methodological considerations in ecological momentary assessment studies on physical activity and sedentary behaviour: a systematic review. Int. J. Behav. Nutr. Phys. Act..

[bb0065] Emerencia A.C., van der Krieke L., Bos E.H., de Jonge P., Petkov N., Aiello M. (2016). Automating vector autoregression on electronic patient diary data. IEEE J. Biomed. Health Inform..

[bb0070] Fisher A.J., Boswell J.F. (2016). Enhancing the personalization of psychotherapy with dynamic assessment and modeling. Assessment.

[bb0075] Fisher A.J., Medaglia J.D., Jeronimus B.F. (2018). Lack of group-to-individual generalizability is a threat to human subjects research. Proc. Natl. Acad. Sci. U. S. A..

[bb0080] Gielissen M.F., Verhagen S., Witjes F., Bleijenberg G. (2006). Effects of cognitive behavior therapy in severely fatigued disease-free cancer patients compared with patients waiting for cognitive behavior therapy: a randomized controlled trial. J. Clin. Oncol..

[bb0085] Gielissen M.F., Verhagen C.A., Bleijenberg G. (2007). Cognitive behaviour therapy for fatigued cancer survivors: long-term follow-up. Br. J. Cancer.

[bb0090] Goedendorp M.M., Peters M.E., Gielissen M.F., Witjes J.A., Leer J.W., Verhagen C.A. (2010). Is increasing physical activity necessary to diminish fatigue during cancer treatment? Comparing cognitive behavior therapy and a brief nursing intervention with usual care in a multicenter randomized controlled trial. Oncologist.

[bb0095] Haslbeck J.M.B., Bringmann L.F., Waldorp L.J. (2020). A tutorial on estimating time-varying vector autoregressive models. Multivar. Behav. Res..

[bb0100] Honaker J., King G., Blackwell M. (2011). Amelia II: a program for missing data. J. Stat. Softw..

[bb0105] Jacobsen P.B., Andrykowski M.A., Thors C.L. (2004). Relationship of catastrophizing to fatigue among women receiving treatment for breast cancer. J. Consult. Clin. Psychol..

[bb0110] Krieke L.V., Jeronimus B.F., Blaauw F.J., Wanders R.B., Emerencia A.C., Schenk H.M. (2016). HowNutsAreTheDutch (HoeGekIsNL): a crowdsourcing study of mental symptoms and strengths. Int. J. Methods Psychiatr. Res..

[bb0115] Lütkepohl H. (2006). New Introduction to Multiple Time Series Analysis.

[bb0120] Mataix-Cols D., Cowley A.J., Hankins M., Schneider A., Bachofen M., Kenwright M. (2005). Reliability and validity of the work and social adjustment scale in phobic disorders. Compr. Psychiatry.

[bb0125] Mohr D.C., Zhang M., Schueller S.M. (2017). Personal sensing: understanding mental health using ubiquitous sensors and machine learning. Annu. Rev. Clin. Psychol..

[bb0130] Molenaar P.C.M., Campbell C.G. (2009). The new person-specific paradigm in psychology. Curr. Dir. Psychol. Sci..

[bb0135] Mundt J.C., Marks I.M., Shear M.K., Greist J.H. (2002). The work and social adjustment scale: a simple measure of impairment in functioning. Br. J. Psychiatry.

[bb0140] Ng M.Y., Weisz J.R. (2016). Annual research review: building a science of personalized intervention for youth mental health. J. Child Psychol. Psychiatry.

[bb0145] Oravecz Z., Tuerlinckx F., Vandekerckhove J. (2016). Bayesian data analysis with the bivariate hierarchical Ornstein-Uhlenbeck process model. Multivar. Behav. Res..

[bb0150] Poort H., Verhagen C.A., Peters M.E., Goedendorp M.M., Donders A.R., Hopman M.T. (2017). Study protocol of the TIRED study: a randomised controlled trial comparing either graded exercise therapy for severe fatigue or cognitive behaviour therapy with usual care in patients with incurable cancer. BMC Cancer.

[bb0155] Prinsen H., Bleijenberg G., Heijmen L., Zwarts M.J., Leer J.W., Heerschap A. (2013). The role of physical activity and physical fitness in postcancer fatigue: a randomized controlled trial. Support Care Cancer.

[bb0160] Prinsen H., van Dijk J.P., Zwarts M.J., Leer J.W., Bleijenberg G., van Laarhoven H.W. (2015). The role of central and peripheral muscle fatigue in postcancer fatigue: a randomized controlled trial. J. Pain Symptom Manag..

[bb0165] Ray C., Weir W., Stewart D., Miller P., Hyde G. (1993). Ways of coping with chronic fatigue syndrome: development of an illness management questionnaire. Soc. Sci. Med..

[bb0170] Rosmalen J.G., Wenting A.M., Roest A.M., de Jonge P., Bos E.H. (2012). Revealing causal heterogeneity using time series analysis of ambulatory assessments: application to the association between depression and physical activity after myocardial infarction. Psychosom. Med..

[bb0175] Ryan O., Kuiper R.M., Hamaker E.L. (2018). A Continuous-time Approach to Intensive Longitudinal Data: What, Why, and How? Continuous Time Modeling in the Behavioral and Related Sciences.

[bb0180] Servaes P., Verhagen C., Bleijenberg G. (2002). Fatigue in cancer patients during and after treatment: prevalence, correlates and interventions. Eur. J. Cancer.

[bb0185] Terwee C.B., Prinsen C.A.C., Chiarotto A., Westerman M.J., Patrick D.L., Alonso J. (2018). COSMIN methodology for evaluating the content validity of patient-reported outcome measures: a Delphi study. Qual. Life Res..

[bb0190] U.S. Department of Health and Human Services NIoH, National Institute of Mental Health (2008). National Institute of Mental Health strategic plan. (DHHS publication no. 08-6368). http://www.nimh.nih.gov/about/strategic-planning-reports/index.shtml.

[bb0195] U.S. Department of Health and Human Services NIoH, National Institute of Mental Health (2015). National Institute of Mental Health Strategic Plan for Research. (DHHS Publication No. 15-6368).

[bb0200] U.S. Department of Health and Human Services NIoH, National Institute of Mental Health (2020). NIMH Strategic Plan for Research (NIH Publication No. 20-MH-8096).

[bb0205] van den Akker L.E., Beckerman H., Collette E.H., Knoop H., Bleijenberg G., Twisk J.W. (2018). Cognitive behavioural therapy for MS-related fatigue explained: a longitudinal mediation analysis. J. Psychosom. Res..

[bb0210] van der Krieke L., Emerencia A.C., Bos E.H., Rosmalen J.G., Riese H., Aiello M. (2015). Ecological momentary assessments and automated time series analysis to promote tailored health care: a proof-of-principle study. JMIR Res. Protoc..

[bb0215] van der Ploeg E., Mooren T.T., Kleber R.J., van der Velden P.G., Brom D. (2004). Construct validation of the dutch version of the impact of event scale. Psychol. Assess..

[bb0220] van Sonderen E. (1993). Het meten van sociale steun met de Sociale Steun Lijst-Interacties (SSL-I) en Sociale Steun Lijst Discrepanties (SSL-D): een handleiding: Noordelijk Centrum voor Gezondheidsvraagstukken (NCGv).

[bb0225] van Sonderen E., Ormel J. (1997). Het meten van aspecten van sociale steun en Hun relatie met welbevinden. een onderzoek naar de bruikbaarheid van de SSL-I ende SSL-D. Gedrag Gezondheid..

[bb0230] Vercoulen J.H.M.M., Swanink C.M.A., Fennis J.F.M., Galama J.M.D., Vandermeer J.W.M., Bleijenberg G. (1994). Dimensional assessment of chronic fatigue syndrome. J. Psychosom. Res..

[bb0235] Vercoulen J.H.M.M., Alberts M., Bleijenberg G. (1999). De checklist individual strength (CIS). Gedragstherapie..

[bb0240] Verhagen S.J.W., Hasmi L., Drukker M., van Os J., Delespaul P.A.E.G. (2016). Use of the experience sampling method in the context of clinical trials. Evid. Based Ment. Health.

[bb0245] Wenze S.J., Miller I.W. (2010). Use of ecological momentary assessment in mood disorders research. Clin. Psychol. Rev..

