# Supplementary material for: Personalizing cognitive behavioral therapy for cancer-related fatigue using ecological momentary assessments followed by automated individual time series analyses: A case report series
Source: Internet Interv. 2021 Jul 14;25:100430. doi: 10.1016/j.invent.2021.100430 (PMC8350606; doi:10.1016/j.invent.2021.100430)
Supplement: The following are the supplementary data related to this article.Supplementary A — Supplement A - EMA survey [file mmc1.docx]

| **Construct** |  | **Item in Dutch** | **Item in English** |
| --- | --- | --- | --- |
| Fatigue | 1 | Ik voelde me moe | I felt tired |
| Depression | 2 | Ik voelde me somber | I felt sad |
| Fear of cancer recurrence | 3 | Ik was bezorgd over of angstig voor de terugkeer van kanker | I was worried or anxious about the recurrence of cancer |
| Physical activity | 4 | Ik was lichamelijk actief. | I was physically active |
| Mental activity | 5 | Ik was mentaal actief (bijvoorbeeld door te lezen, me te concentreren, de administratie te doen). | I was mentally active (for example by reading, concentrating, doing administrative work) |
| Social activity | 6 | Ik was sociaal actief (bijvoorbeeld door te spreken met andere mensen, op bezoek te gaan). | I was socially active (for example by speaking with other people or visiting someone) |
| Focus on fatigue | 7 | Ik dacht veel na over mijn vermoeidheid | I thought a lot about my fatigue |
| Catastrophizing | 8 | Ik was bezig met hoe verschrikkelijk de vermoeidheid aanvoelde | The terrible feel of the fatigue kept me occupied |
| Powerlessness | 9 | Ik voelde me machteloos over mijn vermoeidheid | I felt powerless against my fatigue |
| Self-efficacy | 10 | Ik heb er vertrouwen in dat ik de komende uren de dingen kan doen die ik wil doen | I am confident that I can do the things I want to do in the next few hours |
| Intrusion | 11 | Dingen deden me steeds denken aan kanker en/of de behandeling van kanker | Things kept reminding me of cancer and/or cancer treatment |
| Avoidance | 12 | Ik heb situaties of dingen vermeden die me deden denken aan kanker | I have avoided situations or things that made me think about cancer |
| Lack of social understanding | 13 | Ik stuitte op onbegrip voor mijn vermoeidheid | I was faced with a lack of understanding regarding my fatigue |
